# Supplementary material for: Effect of Magnesium Substitution on Structural Features and Properties of Hydroxyapatite
Source: Materials (Basel). 2023 Aug 30;16(17):5945. doi: 10.3390/ma16175945 (PMC10488744; doi:10.3390/ma16175945)
Supplement: Supplementary file 1 [file materials-16-05945-s001.zip › materials-2499782-supplementary.pdf]

Supplementary Materials

Effect of Magnesium Substitution on Structural Features and Properties of Hydroxyapatite

Vladimir S. Bystrov [1,*], Ekaterina V. Paramonova [1], Leon A. Avakyan [2], Natalya V. Eremina [3], Svetlana V. Makarova [3] and Natalia V. Bulina [3]

[1] Institute of Mathematical Problems of Biology—Branch of Keldysh Institute of Applied Mathematics, Russian Academy of Sciences, 142290 Pushchino, Russia; ekatp11@gmail.com
[2] Physics Faculty, Southern Federal University, 344090 Rostov-on-Don, Russia; laavakyan@sfdeu.ru
[3] Institute of Solid State Chemistry and Mechanochemistry, Siberian Branch, Russian Academy of Sciences, 630128 Novosibirsk, Russia; eremina@solid.nsc.ru (N.V.E.); makarova@solid.nsc.ru (S.V.M.); bulina@solid.nsc.ru (N.V.B.)
* Correspondence: vsbys@mail.ru or bystrov@impb.ru

S1. Initial data for models. Selected initial positions of atoms for substitution modeling:

Table S1.1. Substitutions of Ca atoms for Mg atoms in Ca1 positions (nMg/Ca1) in the supercell.

#	Atom # in Ca1	x	y	z
1	#1	0.003	10.9456	0.026
2	#5	9.4939	10.9484	0.0154
3	#17	-0.0085	5.4666	3.4425
4	#21	9.4787	5.473	3.4399
5	#26	-0.0025	10.9466	10.27
6	#30	9.4697	10.9507	10.2882
7	#10	0.0039	5.472	13.7121
8	#14	9.4745	5.4736	13.7122
9	#32	14.2301	2.7377	10.2842
10	#16	14.2417	13.6882	13.7068
11	#12	4.7432	13.6636	13.7128
12	#28	4.7231	2.7571	10.2996
13	#7	14.2201	2.7406	0.0161
14	#23	14.232	13.6548	3.4348
15	#19	4.7058	13.6827	3.4412
16	#3	4.7491	2.7299	0.0261

Table S1.2. Substitutions of Ca atoms for Mg atoms in Ca2 positions (nMg/Ca2) in the supercell.

#	Atom # in Ca2	x	y	z
1	#33	1.1684	14.3487	1.7162
2	#51	8.2811	14.3733	5.1552
3	#65	2.3644	8.2347	1.7199
4	#69	11.8558	8.2288	1.7258
5	#34	1.172	14.383	8.5738
6	#52	8.2824	14.3823	12.0156
7	#66	2.3857	8.2171	8.5767
8	#70	11.8469	8.226	8.5841
9	#48	17.7807	2.0859	12.0086
10	#80	16.6355	8.2043	12.0045
11	#76	7.1329	8.1959	12.0109
12	#44	8.3078	2.044	12.014
13	#47	17.7976	2.0649	5.1464
14	#79	16.5973	8.2056	5.1395
15	#75	7.1069	8.1892	5.1539
16	#43	8.3085	2.055	5.1583

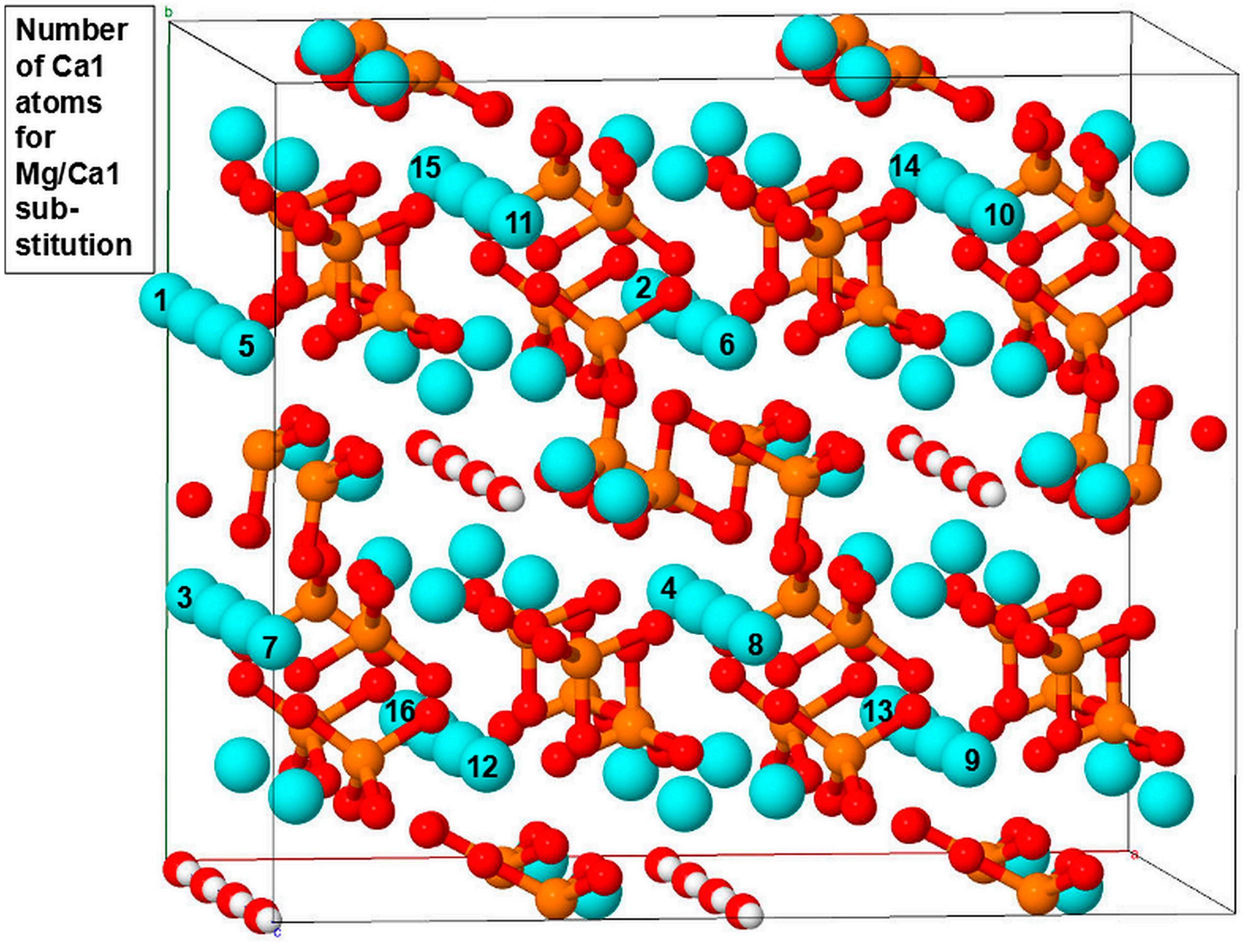

Figure S1.1. Initial models of the HAP supercell of 352 atoms with the indicated selected positions of atoms for replacing the Ca atom with Mg atoms with marked numbers from 1 to 16 in the positions of the Ca1.

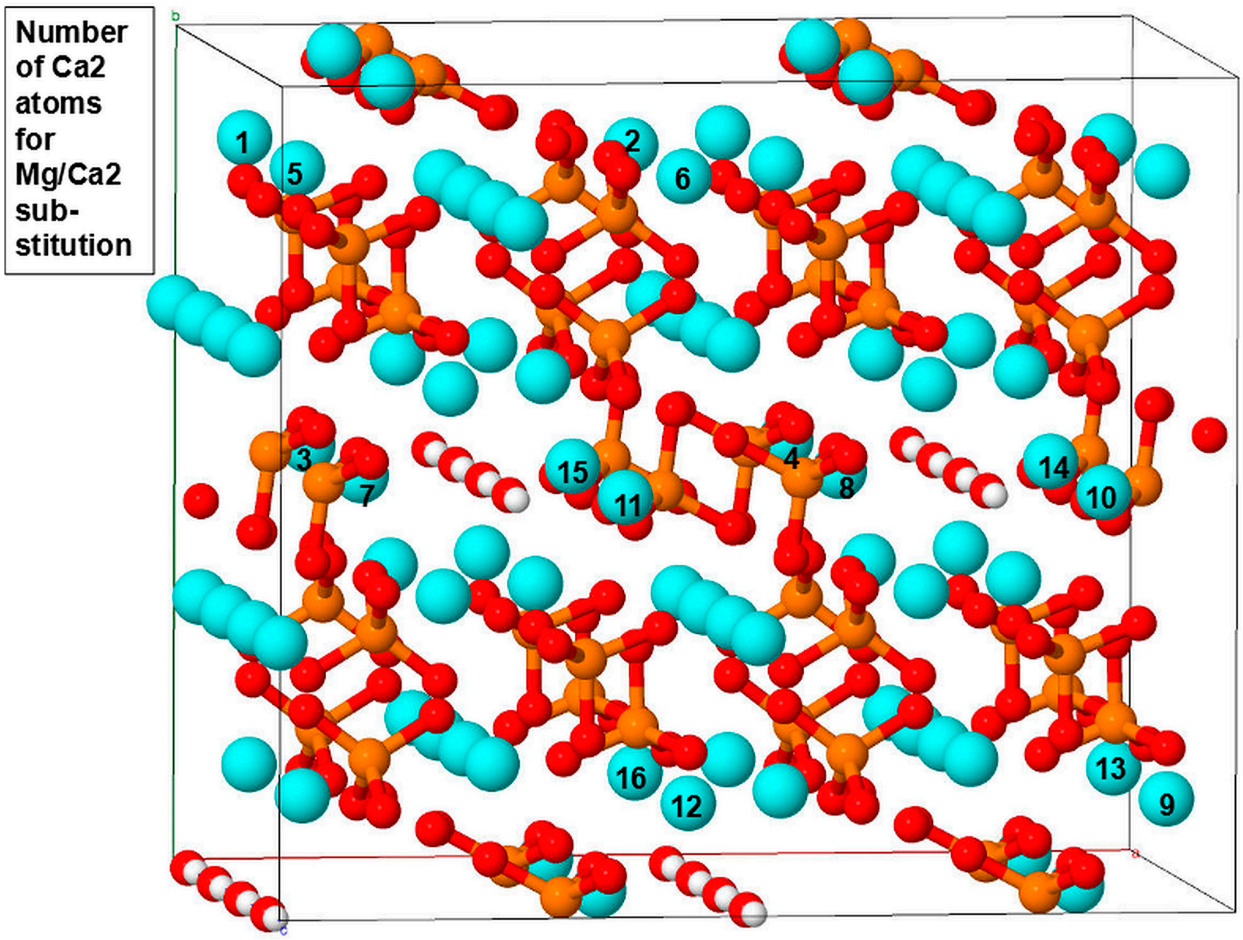

Figure S1.2. Initial models of the HAP supercell of 352 atoms with the indicated selected positions of atoms for replacing the Ca atom with Mg atoms with marked numbers from 1 to 16 in the positions of the Ca2.

S2. Main data obtained by the DFT calculation results:

Table S2.1. Computed unit cell parameters and volume for HAP-Mg supercell with different Mg content in different positions (Ca1 and Ca2). n – number of Mg atoms in supercell; x – number of Mg atoms in unit cell.

Mg content		Ca1 substitution				Ca2 substitution			
n	x	a (Å)	b (Å)	c (Å)	V (Å^3)	a (Å)	b (Å)	c (Å)	V (Å^3)
0	0	9.4812	9.4812	6.8586	533.948	9.4812	9.4812	6.8586	533.948
1	0.125	9.4775	9.4755	6.8513	532.844	9.4815	9.4721	6.8518	532.912
2	0.25	9.4722	9.4720	6.8445	531.820	9.4780	9.4696	6.8445	532.007
4	0.5	9.4692	9.4657	6.8302	530.191	9.4640	9.4674	6.8315	530.090
8	1.0	9.4412	9.4412	6.8198	526.349	9.4344	9.4392	6.8205	526.008
12	1.5	9.4062	9.4109	6.8049	521.668	9.4238	9.4232	6.7919	522.332
16	2.0	9.4022	9.3965	6.7797	518.732	9.4018	9.3961	6.7814	518.257
		Ca1 substitution with one OH vacancy				Ca2 substitution with one OH vacancy			
0	0	9.4799	9.4778	6.8578	533.608	9.4799	9.4778	6.8578	533.608
4	0.5	9.4643	9.4641	6.8307	529.861	9.4658	9.4633	6.8325	530.038
16	2.0	9.3891	9.3910	6.7962	518.956	9.4029	9.3828	6.7816	518.152

* Estimated errors: $\Delta a = \Delta b \approx 0.005$ Å, $\Delta c \approx 0.002$ Å per unt cell.

Table S2.2. Distances r from H atom of OH group to atoms in Ca1 positions at different amount of Mg atoms in Ca1 positions of supercell with nMg/Ca1 substitution, where n is a number of Mg atoms in supercell.*)

Atom numbers		r(H – Ca1) or r(H-Mg/Ca1), Å				
H	Ca1	0Mg/Ca1, x = 0	4Mg/Ca1, x = 0.5	8Mg/Ca1, x = 1	12Mg/Ca1, x = 1.5	16Mg/Ca1, x = 2
Left OH-channel						
#340	#12	5.49301	5.29125	5.43029	5.23945	5.23606
#348	#30	5.48226	5.34166	5.20923	5.20576	5.25317
#340	#14	5.47884	5.35986	5.35027	5.28078	5.23682
#348	#28	5.50115	5.29419	5.17634	5.18851	5.30286
#340	#10	5.50908	5.45668	5.37989	5.32839	5.28998
#348	#26	5.49519	5.31751	5.15959	5.14081	5.27768
Right OH-channel						
#344	#16	5.496925	5.291107	5.23899	5.24347	5.25312
#352	#30	5.502718	5.35919	5.39027	5.35029	5.30620
#344	#14	5.478873	5.45700	5.37998	5.35633	5.28949
#352	#32	5.505023	5.27513	5.23997	5.20177	5.27153

*) Hydrogen atoms #340 and #344 lie approximately in the same 1st section plane with calcium or magnesium atoms in positions Ca1 #10, #12, #14, #16 with coordinate z = 13.25 – 13.71 Å; while the hydrogen atoms #348 and #352 lie in the 2nd section plane together with the calcium or magnesium atoms #26, #28, #30, #32 in Ca1 positions with coordinates z = 9.79 – 10.24 Å.

Table S2.3. Distances r from oxygen atom of OH group to Ca atoms in Ca2 at different amount of Mg atoms in Ca1 positions of supercell with nMg/Ca1 substitution, where n is a number of Mg atoms in supercell.*)

Atom numbers		r(O-Ca2), Å				
O	Ca2	0Mg/Ca1, x = 0	4Mg/Ca1, x = 0.5	8Mg/Ca1, x = 1	12Mg/Ca1, x = 1.5	16Mg/Ca1, x = 2
Left OH-channel						
#324	#42	2.37823	2.35171	2.35445	2.33695	2.34292
#332	#60	2.37756	2.31117	2.29296	2.26832	2.30023
#324	#76	2.38342	2.32137	2.33879	2.32616	2.34904
#332	#36	2.37683	2.31117	2.28723	2.28309	2.29300
#324	#50	2.38256	2.31424	2.32879	2.32180	2.34599
#332	#66	2.38470	2.32564	2.28389	2.26352	2.30329
Right OH-channel						
#328	#46	2.38361	2.35158	2.35449	2.33602	2.34137
#336	#64	2.38197	2.30578	2.29292	2.26729	2.29936
#328	#80	2.37963	2.32136	2.33874	2.32599	2.33937
#336	#40	2.38410	2.32263	2.28725	2.28291	2.29750
#328	#54	2.38514	2.31420	2.33879	2.32901	2.35596
#336	#70	2.37512	2.32562	2.28391	2.26289	2.29241

*) Oxygen atoms O #324 and #328 lie approximately in the same 1st section plane with calcium or magnesium atoms in Ca2 positions #42, #50, #76, (left OH-channel) and # 46, #54, # 80 (right OH-channel) with coordinates z = 12.00 – 12.27 Å; while oxygen atoms O #332 and #336 lie in the 2nd section plane with calcium or magnesium atoms #36, #60, #66 (left OH-channel) and #40, #64, #70 (right OH-channel) in Ca2 positions with coordinates z = 8.58 – 8.82 Å.

Table S2.4. Distances r between atoms in Ca1 positions with numbers #28, #30, #32 and the nearest oxygen atoms of PO_4 groups in dependence of amount of Mg atoms at the substitution nMg/Ca1.

Atom numbers		r(Mg/Ca1 – O), Å							
Mg/Ca1	O	r_0, 0Mg/Ca1	8Mg/Ca1, x = 1	Δr = = r_{8Mg} - r_0	Δr/r_0, %	12Mg/Ca1, x = 1.5	16Mg/Ca1, x = 2	Δr = = r_{16Mg} -r_0	Δr/r_0, %
Around #28				0 – 10% Mg				0 – 20% Mg	
#28	162	2.38259	2.30992	0.07267	3.05	2.11379	2.14551	0.23708	9.95
#28	196	2.42674	2.36654	0.0602	2.48	2.10533	2.05433	0.37241	15.35
#28	156	2.38776	2.37504	0.01272	0.53	2.16161	2.15254	0.23522	9.85
#28	186	2.42651	2.34359	0.08292	3.42	2.10367	2.05084	0.37567	15.48
#28	130	2.38447	2.34110	0.04337	1.82	2.14609	2.16196	0.22251	9.33
#28	218	2.43449	2.40299	0.0315	1.29	2.13715	2.05645	0.37804	15.52
Around #30									
#30	164	2.38019	2.19707	0.18312	7.7	2.16631	2.12986	0.25033	10.5
#30	198	2.42795	2.24326	0.18469	7.6	2.05679	2.05868	0.36927	15.2
#30	158	2.39117	2.301	0.09017	3.8	2.12229	2.15357	0.2376	9.9
#30	188	2.41933	2.14466	0.27467	11.4	2.06361	2.03334	0.38599	16.0
#30	132	2.38921	2.25585	0.13336	5.6	2.18763	2.17896	0.21025	8.8
#30	220	2.43629	2.09906	0.33723	13.84	2.04275	2.05904	0.37725	15.5
Around #32									
#32	166	2.38448	2.30988	0.0746	3.13	2.11965	2.13916	0.24532	10.29
#32	200	2.42435	2.36653	0.0578	2.38	2.09901	2.05586	0.36849	15.20
#32	160	2.38354	2.37505	0.0085	0.36	2.16209	2.15863	0.22491	9.44
#32	190	2.42489	2.34346	0.0814	3.36	2.1031	2.04760	0.37729	15.60
#32	134	2.38457	2.34119	0.0434	1.82	2.15066	2.15920	0.22537	9.45
#32	222	2.4228	2.40599	0.0168	0.69	2.13969	2.05629	0.36651	15.13

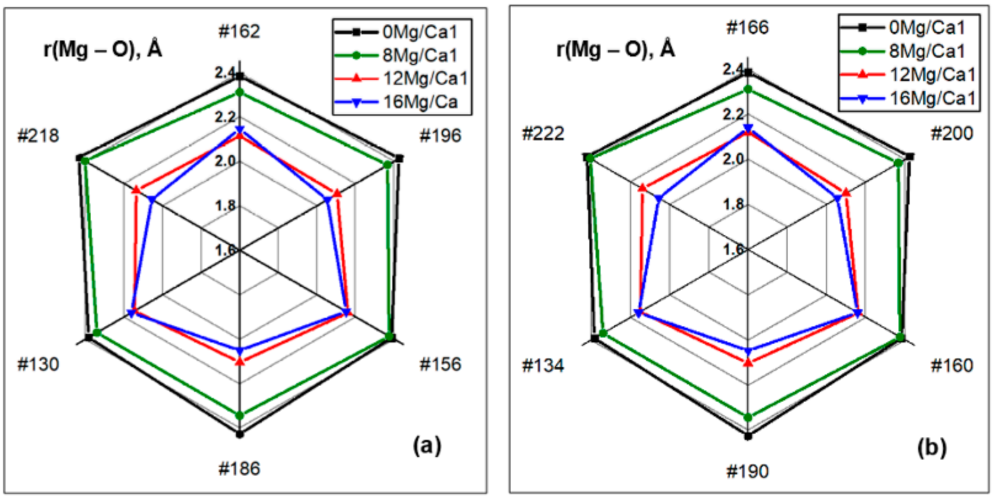

Figure S2.1. Diagrams of distances r(Ca1/Mg–O) depending on the amount of substituent (n) upon substitution nMg/Ca1: a) Mg #28; b) Mg#32.

Table S2.5. Distances r between atoms in Ca2 positions to O atoms in the OH-channel of Ca and Mg atoms in Ca2 position to the oxygen from OH group in dependence of amount of Mg atoms at the substitution nMg/Ca1.*)

Atom numbers		r(O–Ca2), Å			
O	Ca2	0Mg/Ca1, x = 0	8Mg/Ca1, x = 1	12Mg/Ca1, x = 1.5	16Mg/Ca1, x = 2
Left OH-channel					
#324	#42	2.42219	2.30794	2.39424	2.39232
#332	#60	2.41301	2.41282	2.41749	2.40501
#324	#76	2.31894	2.2888	2.08323	2.0803
#332	#36	2.42077	2.4034	2.40767	2.39388
#324	#50	2.41832	2.32669	2.40977	2.40162
#332	#66	2.31858	2.08125	2.08557	2.08224
Right OH-channel					
#328	#46	2.42792	2.30753	2.39495	2.38934
#336	#64	2.41806	2.37853	2.40006	2.38782
#328	#80	2.31144	2.29482	2.08659	2.08209
#336	#40	2.42764	2.3995	2.39543	2.37823
#328	#54	2.41897	2.32563	2.39074	2.38836
#336	#70	2.30902	2.10253	2.09402	2.0885

*) Oxygen atoms O #324 and #328 lie approximately in the same 1st section plane with calcium or magnesium atoms in Ca2 positions #42, #50, #76, (left OH-channel) and # 46, #54, # 80 (right OH-channel) with coordinates z = 12.00 – 12.27 Å; while oxygen atoms O #332 and #336 lie in the 2nd section plane with calcium or magnesium atoms #36, #60, #66 (left OH-channel) and #40, #64, #70 (right OH-channel)) in Ca2 positions with coordinates z = 8.82 – 8.58 Å.

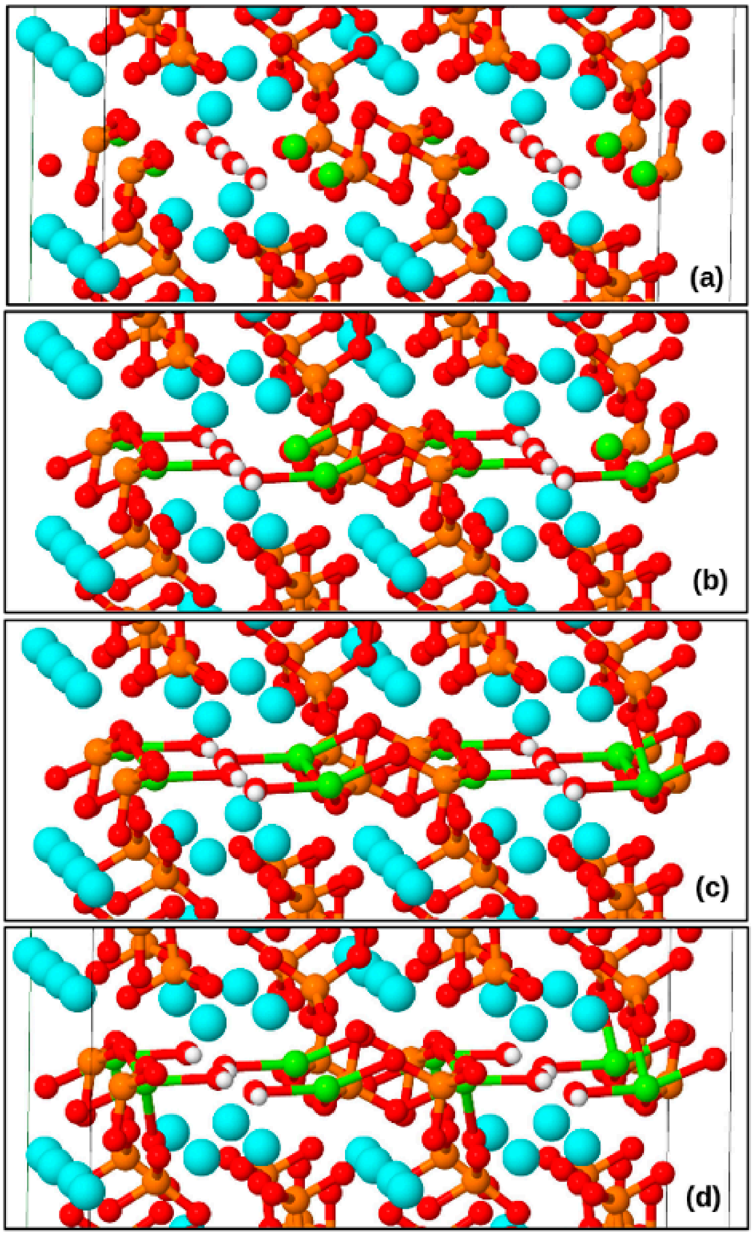

Figure S2.2. Successive stages of relaxation of the 16Mg/Ca2 supercell with two OH channels with Mg concentration x = 2: a – initial state; b – appearance of the first few Mg–O ionic bonds after several stages of relaxation; c – addition of new Mg–O ionic bonds with continued relaxation; d – final shifts of Mg–O bound ions in different directions inside the OH channel upon completion of relaxation.

Table S2.6. Changes in the band gap Eg and Eg*, as well as the energies E_1 and E_2 with increasing Mg concentration and in the presence of an OH-vac vacancy. The relative changes in these energies at different concentrations x of magnesium Mg are also given.

Mg-content		nMg/Ca1				nMg/Ca2			
n	x	Eg, PBE, eV	ΔEg, PBE, eV	Eg, HSE, eV	ΔEg, HSE, eV	Eg, PBE, eV	ΔEg, PBE, eV	Eg, HSE, eV	ΔEg, HSE, eV
0	0	5.34	0	7.05	0	5.37	0	7.08	0
1	0.125	5.64	0.30	7.39	0.34	5.61	0.25	7.37	0.28
2	0.25	5.65	0.31	7.40	0.35	5.61	0.24	7.36	0.28
4	0.5	5.65	0.31	7.40	0.35	5.57	0.20	7.32	0.24
8	1.0	5.74	0.40	7.58	0.48	5.53	0.16	7.28	0.20
12	1.5	5.73	0.41	7.61	0.56	5.48	0.11	7.23	0.15
16	2.0	5.78	0.44	7.59	0.54	5.43	0.06	7.19	0.10
		nMg/Ca1 - 1OH				nMg/Ca2 - 1OH			
		Eg*, PBE, eV	ΔEg*, (ΔEg**), PBE, eV	Eg*, HSE, eV	ΔEg*, (ΔEg**), HSE, eV	Eg*, PBE, eV	ΔEg*, (ΔEg**), PBE, eV	Eg*, HSE, eV	ΔEg*, (ΔEg**), HSE, eV
0	0	5.69	0.35 (0)	7.45	0.40 (0)	5.69	0.33 (0)	7.45	0.37 (0)
4	0.5	5.69	0.35 (0.043)	7.41	0.36 (0.01)	5.65	0.29 (0.09)	7.41	0.33 (0.09)
16	2.0	5.51	0.17 (-0.27)	7.28	0.23 (-0.31)	5.55	0.18 (0.11)	7.30	0.22 (0.12)
		E_1, PBE, eV	ΔE_1, (ΔE_1*), PBE, eV	E_1, HSE, eV	ΔE_1, (ΔE_1*), HSE, eV	E_1, PBE, eV	ΔE_1, (ΔE_1*), PBE, eV	E_1, HSE, eV	ΔE_1, (ΔE_1*), HSE, eV
0	0	4.94	-0.40 (-0.75)	5.36	-1.68 (-2.08)	4.94	-0.43 (-0.75)	5.36	-1.72 (-2.08)
4	0.5	4.30	-1.35 (-1.40)	5.37	-1.68 (-2.04)	4.08	-1.49 (-1.58)	5.37	-1.95 (-2.04)
16	2.0	4.35	-1.43 (-1.16)	5.70	-1.35 (-1.58)	3.96	-1.47 (-1.59)	5.25	-1.94 (-2.06)
		E_2, PBE, eV		E_2, HSE, eV		E_2, PBE, eV		E_2, HSE, eV	
0	0	0.75		2.08		0.75		2.08	
4	0.5	1.40		2.04		1.58		2.04	
16	2.0	1.16		1.58		1.59		2.06	

Note: Here are the following changes in the calculated energies of the electronic levels, respectively:

$$\Delta Eg = Eg(x) – Eg(x=0)\ ;\ \Delta Eg^* = Eg^*(x) – Eg(x=0);\ \Delta Eg^{**} = Eg^*(x) – Eg^*(x=0);$$

$$\Delta E_1 = E_1(x) – Eg(x=0);\ \Delta E_1^* = E_1(x) – Eg^*(x=0);\ \ E_2 = Eg^*(x) — E_1(x)$$

S3. Some data obtained by the experimental studies.

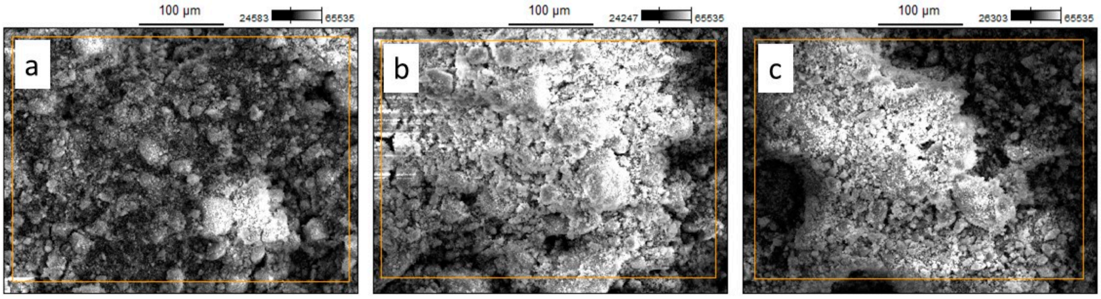

Figure S3.1. SEM images of regions for EDX microanalysis: a – sample with x=0; b – sample with x=1; c – sample with x=2.

Table S3.1. Expected and measured concentration (wt%) of elements in synthesized HAP-Mg samples. Energy dispersive X-ray semyquantitative microanalysis of regions presented in Figure S3.1.

		x=0			x=1			x=2	
	Mg	P	Ca	Mg	P	Ca	Mg	P	Ca
Expected	0	31.7	68.3	4.2	32.6	63.2	8.8	33.5	57.8
Measured	0	29	71	4	28	68	9	30	61
